# TRPA1-mediated repellency behavior in the red flour beetle *Tribolium castaneum*

**DOI:** 10.1038/s41598-022-19580-z

**Published:** 2022-09-10

**Authors:** Kenji Shimomura, Soshi Ino, Kazuya Tamura, Takehito Terajima, Motohiro Tomizawa

**Affiliations:** Department of Chemistry for Life Sciences and Agriculture, Tokyo University of Agriculture, Sakuragaoka 1-1-1, Setagaya-ku, Tokyo 156–8502 Japan

**Keywords:** Entomology, RNAi

## Abstract

The sensory perception of irritant chemicals results in escape and repellency behavioral patterns in insects. Transient receptor potential channels are cation channels that function as sensor proteins for several types of signals, such as light, sound, temperature, taste, as well as chemical and physical stimuli; among these, the TRPA channel is widely conserved and activated by irritant chemicals. Certain plant-derived essential oils (EOs), produced by secondary metabolism, are mixtures of volatile compounds, which are used as repellents because they contain environmentally sustainable ingredients. Citronellal, which is present in citronella EO from *Cymbopogon* species, is a potentially viable insect repellent; however, the repellency capability against coleopteran beetles remains limited. We investigated the citronellal-derived repellency behavior for the red flour beetle *Tribolium castaneum*, in which TcTRPA1 and odorant receptor co-receptor (Orco) expressions were mediated by RNA interference. Area-preference tests showed dose-dependent repellency behavior for citronellal; additionally, both *TcTRPA1* and *TcOrco* double-strand RNA (dsRNA) micro-injection showed clear *TcTRPA1* and *TcOrco* transcript reductions, and only *TcTRPA1* dsRNA treatment significantly impaired repellency behavior. The relative expression level of the *TcTRPA1* transcripts, evaluated by quantitative reverse-transcription PCR (qRT-PCR), revealed dominant expression in the antennae, indicating the antennae-expressed TcTRPA1-mediated repellency behavior.

## Introduction

Nociception refers to the sensory perception of a noxious stimulus that has the potential to cause pain and/or damage, resulting in escape and repellency behavioral patterns^1^. Several types of stimuli trigger nociceptive sensory transduction, for instance, heat, mechanical stimulation, and chemicals.

Multiple types of membrane proteins, receptors, and ion channels participate in stimulus detection. Transient receptor potential (TRP) channels are cation channels evolutionarily conserved between vertebrates and invertebrates^2^. TRP channels function as the primary sensors for various types of information, including light, sound, temperature, taste, and chemical and physical stimuli. The TRP family is divided into seven subfamilies, namely TRPC, TRPA, TRPV, TRPN, TRPM, TRPP, and TRML, which are based on the constituent sequence elements^3^.

The TRPA channel is widely conserved and contains multiple ankyrin repeats in the N-terminal region^4^. TRPA1 is activated by nociceptive thermal and irritant chemicals, such as noxious odorants and tastants. In mammals, TRPA1 is expressed in a subset of nociceptive neurons, and TRPA1 functions as a chemical nocisensor for numerous chemicals, such as pungent natural compounds, allyl isothiocyanate (AITC), menthol, and cinnamaldehyde^5,6^. The chordate TRPA family contains only one member, namely TRPA1, while *Drosophila melanogaster* genome contains four TRPA subfamily genes: *TRPA1* which is the chordate *TRPA1* homologue, *water witch*, *pyrex*, and *painless*. An additional TRPA subfamily gene, *TRPA5*, is present in the coleopteran model insect, the red flour beetle *Tribolium castaneum* genome, and although hymenopteran genomes lack *TRPA1*, they contain *TRPA5* and the Hymenoptera-specific *TRPA* (*HsTRPA*) genes^7^. In the other subgroups, the TRPM channel was initially named melastatin protein, as it was identified from melanocytes^8,9^; furthermore, the mammalian TRPM8 channel has been reported to be activated by a chemical stimulus, namely menthol and its analogous compounds^10^. Although the TRPM subfamily consists of eight channels (TRPM1–8), only one TRPM channel gene is present in insects^7^. Intriguingly, certain naturally occurring ligands have been shown to activate multiple TRP channels; for example, menthol activates TRPA1 and TRPM8 in mammals^11,12^.

Plant-derived essential oils (EOs) are mixtures of volatile compounds used as insecticides, repellents, and oviposition deterrents, as they contain environmentally sustainable ingredients produced by secondary metabolism^13,14^. Among the EOs, citronella oil, extracted from *Cymbopogon* species, is a potentially powerful insect repellent against several species of mosquitoes, booklouse, and beetle^15–18^. Among the chemical components, a monoterpene compound citronellal, 3,7-dimethyl-6-octenal, is one of the major compounds driving the insecticidal activity and repellency behavior for *T. castaneum*^16,19^.

Prior studies have been conducted on the molecular recognition mechanism of citronellal activity, in which insect TRPA1 is necessary to repel citronellal in two dipteran species, *D. melanogaster* and *Anopheles gambiae* mosquito. It is noteworthy that citronellal activated both TRPA1s when they were heterologously expressed in *Xenopus* oocytes^20,21^.

Alternatively, odorant perception derived from host plants is executed by peripheral odorant receptors (ORs) that are expressed on the dendrites of olfactory sensory neurons (OSNs)^22,23^. Insect ORs form heteromeric cation channels with the obligate odorant receptor co-receptor (Orco), which functions not only as a measure of odorant sensitivity but also as a chaperone for the localization and maintenance of ORs^24^. Therefore, *Orco* knockdown and/or null mutations result in critical olfactory loss of function^25^.

In *Drosophila*, two pathways are involved in the citronellal response^20^. One pathway is mediated by OR reception, because *Orco* mutation resulted in impaired repellent activity; the other pathway is the G-protein-coupled phospholipase C signaling cascade, in which the TRPA1 channel functions downstream, and the TRPA1 isoform, namely TRPA1(A), elicited citronellal-enhanced gustatory aversion^21,26^. Additionally, the TRPA1 isoform, namely TRPA1s, in mosquito species such as *A. gambiae* and *Culex pipiens*, is directly activated by citronellal^20,27^. It was described several TRPA1 isoforms in other insect species, to date, there are no reports about TRPA1 isoforms in *T. castaneum*.

In related to TRPA1 mediated chemoreception, it was reported that menthol, extracted from *Mentha* species, evokes nocifensive rolling behavior in *Drosophila* larvae, and that TRPA1 and TRPM are genetically interact and required for the manifestation of aversive behavior^28^. In vitro expression analysis showed that *Drosophila* TRPA1 was not activated by menthol^6^, underscoring the continuing debate on whether TRPA1 functions in the perception of menthol.

However, the repellency mechanism of citronellal against coleopteran beetles is yet to be elucidated. In addition, it remains unclear whether menthol stimulates the TRPA1-mediated repellency behavior of *T. castaneum*, although we previously reported that *l*-menthol evokes TcTRPM-mediated contact repellency behavior in *T. castaneum*^29^. The objective of the present study was to examine the repellency behavior of *T. castaneum* to citronellal using RNAi-mediated knockdown of *TcTRPA1*; this was aimed at determining whether TcTRPA1 participates in mediating the repellency response. We further examined the OR-mediated repellency behavior for citronellal and clarified the engagement of TcTRPA1 for menthol-derived repellency behavior in combination with TcTRPM.

## Results

### Repellency behavior for citronellal

**Figure 1:**
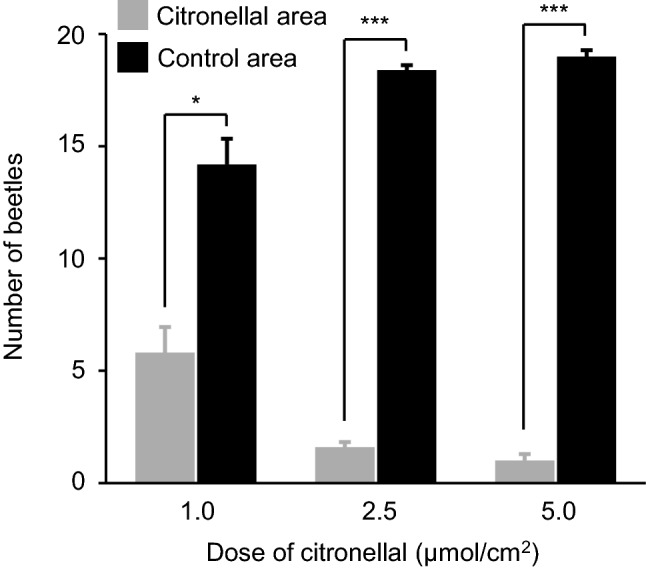
Dose-dependent repellency behavior of *Tribolium castaneum* for citronellal. Twenty beetles were released in the area-preference test: in each dose, five biological replications were performed. Data are expressed as mean ± standard error of the mean; asterisks above the bars indicate significant differences between the number of beetles on the citronellal-treated area and on the control area (only solvent applied) (paired t-test, * *P* < 0.05, *** *P* < 0.001).

The area-preference test revealed a dose-dependent repellency activity of citronellal against *T. castaneum* (Fig. 1). Citronellal showed high repellency activity at more than 2.5 μmol/cm^2^. Based on these results, we decided to set the concentration at 2.5 μmol/cm^2^ for further analysis.

To clarify citronellal target, we cloned *TcTRPA1* gene, and the full-length amino acid sequence showed high sequence identities (74–83%) with some coleopteran TRPA1s. The phylogenetic tree revealed a cluster in the coleopteran TRPA1s among insect TRPA1 channels (Supplementally Fig. S1).

**Figure 2:**
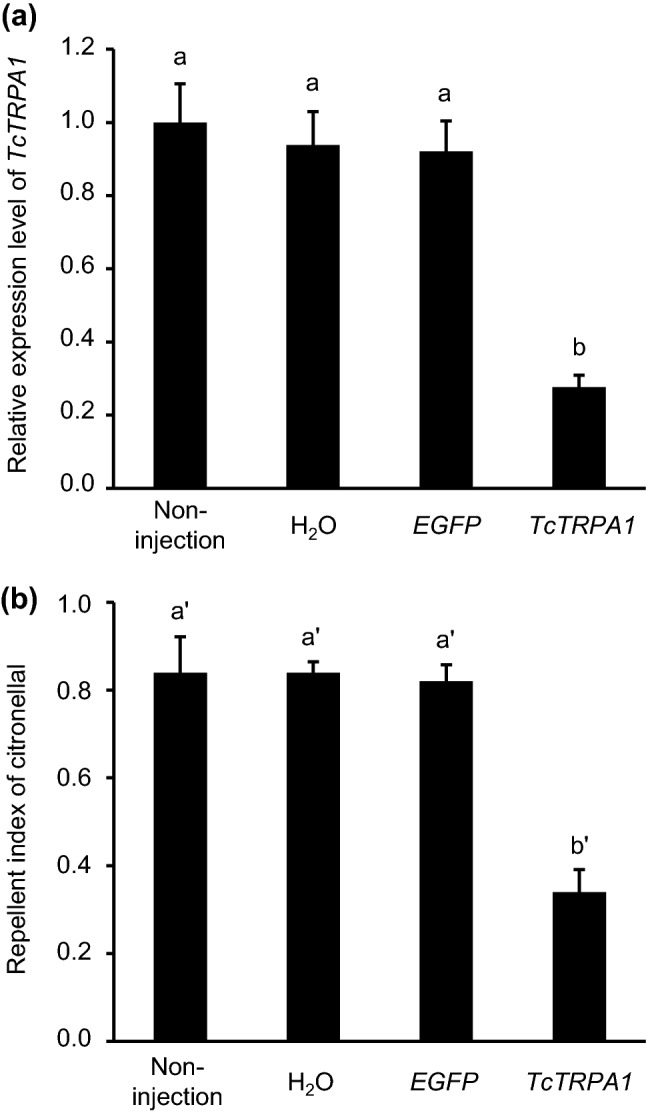
RNAi-mediated *TcTRPA1* knockdown resulted in hampered citronellal repellency behavior. (**a**) *TcTRPA1* dsRNA was micro-injected into the pupae; after the adult emerged, the targeted transcripts were measured by qRT-PCR. The expression level was revealed as fold change relative to the expression level seen in the non-injected beetles (n = 3). (**b**) The mean repellent indices for citronellal were compared in the area-preference test (n = 5). Data are expressed as mean ± standard error of the mean. The same letters above the bars indicate no significant difference at *P* > 0.05 (one-way ANOVA and Tukey–Kramer HSD tests). TRP: transient receptor potential.

Target transcripts suppression by RNAi was evaluated using quantitative reverse-transcription PCR (qRT-PCR). The *TcTRPA1* double-stranded RNA (dsRNA) treatment successfully suppressed target *TcTRPA1* transcripts (*F*_3,8_ = 57.26, *P* < 0.001). The qRT-PCR analysis showed a reduction in the target *TcTRPA1* transcripts to 27% (Fig. 2a). *TcTRPA1* transcripts of solvent (H_2_O) and *enhanced green fluorescent protein* (*EGFP*) dsRNA treatments were not significantly different from those of non-injected beetles.

Behavioral studies of beetles treated with *TcTRPA1* dsRNA revealed significantly altered repellency behavior. *TcTRPA1* dsRNA injection significantly hampered the repellency behavior for citronellal (*F*_3,16_ = 14.26, *P* < 0.001) (Fig. 2b). The repellency activities of the beetles injected with solvent or *EGFP* dsRNA were not significantly different from those of non-injected beetles.

The *TcTRPA1* dsRNA-injected beetles were compared for standard synthetic repellent, *N*,*N*-diethyl-3-methylbenzamide (DEET) using the area-preference test. The beetles treated with the *TcTRPA1* dsRNA showed repellency behavior for DEET, which was not significantly different from that of non-injected beetles (t-test, *P* = 0.084) (Supplementary Fig. S2).

### Relative expression level of *TcTRPA1* transcripts and antenna involvement in the repellency behavior

**Figure 3:**
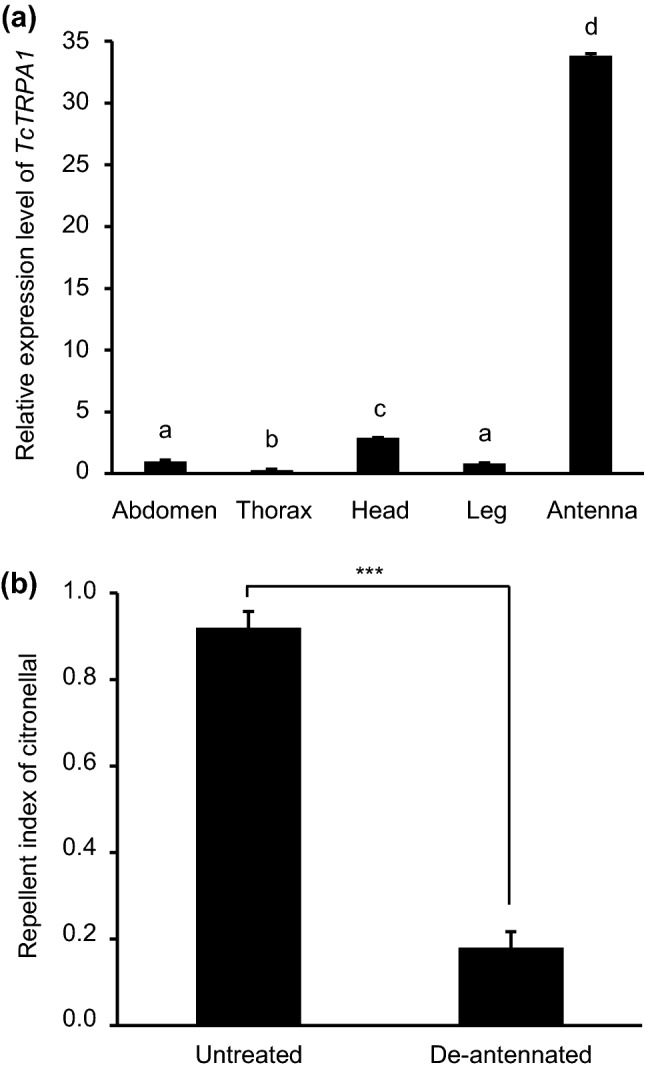
Tissue expression profile of *TcTRPA1* and antenna involvement in the repellency behavior. (**a**) Relative expression levels of *TcTRPA1* transcripts in each abdomen, thorax, head, leg, and antenna specimen of adult *Tribolium castaneum* were revealed as fold change relative to the corresponding transcript levels seen in the abdomen (n = 3) analyzed by qRT-PCR. Data are expressed as mean ± standard error of the mean (SEM). The same letters above the bars indicate no significant difference at *P* > 0.05 (one-way ANOVA and Tukey–Kramer HSD tests). (**b**) Mean repellent index of untreated and de-antennated adult beetles treated with 2.5 μmol /cm^2^ of citronellal (n = 5). Data are expressed as mean ± SEM, and *** indicates that there was significant difference between them (*** *P* < 0.001, two-tailed student’s t-test). TRP: transient receptor potential.

The relative expression profiles of the *TcTRPA1* transcripts in different tissues were analyzed by qRT-PCR. *TcTRPA1* was predominantly expressed in the antennae (*F*_4,10_ = 337.00, *P* < 0.001) (Fig. 3a), and the transcript level was almost 34 times higher than that in the abdomen.

To elucidate antennae-based citronellal detection by TcTRPA1, an area-preference test was performed on the de-antennated beetles. The results showed that the de-antennated beetles had a significantly reduced repellent index compared with the repellent index in the untreated beetles (t-test, *P* < 0.001) (Fig. 3b).

### RNAi-mediated knockdown of *TcOrco*

**Figure 4:**
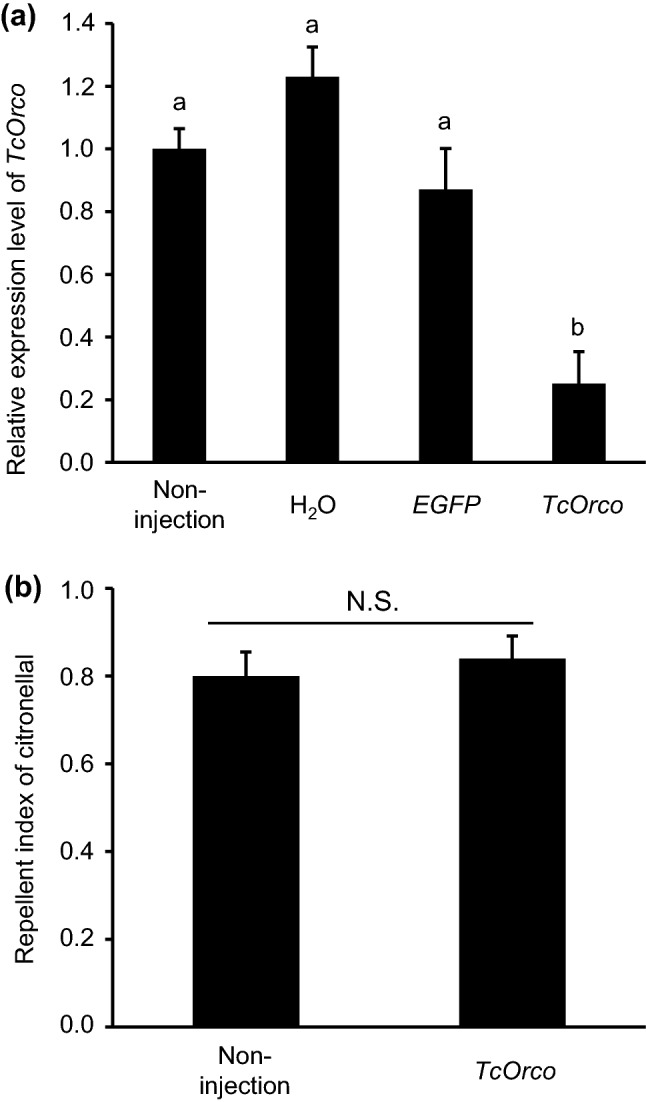
TcOrco did not mediate the citronellal-based repellency behavior of *Tribolium castaneum*. (**a**) *TcOrco* dsRNA was micro-injected into the pupae; after the adult emerged, the targeted transcripts were measured by qRT-PCR. The expression level was revealed as fold change relative to the expression level seen in the non-injection beetles (n = 3). Data are expressed as mean ± standard error of the mean (SEM). The same letters above the bars indicate no significant difference at *P* > 0.05 (one-way ANOVA and Tukey–Kramer HSD tests). (**b**) The mean repellent indices for citronellal were compared in the area-preference test (n = 5). Data are expressed as mean ± SEM, and N.S. indicates there was no significant difference between non-injected and *TcOrco* dsRNA treated beetles (*P* = 0.608, two-tailed student’s t-test). Orco: odorant receptor co-receptor.

The *TcOrco* dsRNA treatment was performed for further analysis of antenna-based citronellal detection specificity. The treated beetles showed a reduction in the target *TcOrco* transcripts to 25% (*F*_3,8_ = 53.94, *P* < 0.001) based on the qRT-PCR results (Fig. 4a); the subsequent area-preference test showed that the repellency activity of citronellal was not significantly different from that of untreated beetles (t-test, *P* = 0.608) (Fig. 4b).

The relative expression profile of the *TcOrco* transcripts among different tissues confirmed that *TcOrco* was predominantly expressed in the antennae (*F*_4,10_ = 350.60, *P* < 0.001) (Supplementary Fig. S3).

### Intervention of TcTRPA1 for *l*-menthol repellency

**Figure 5:**
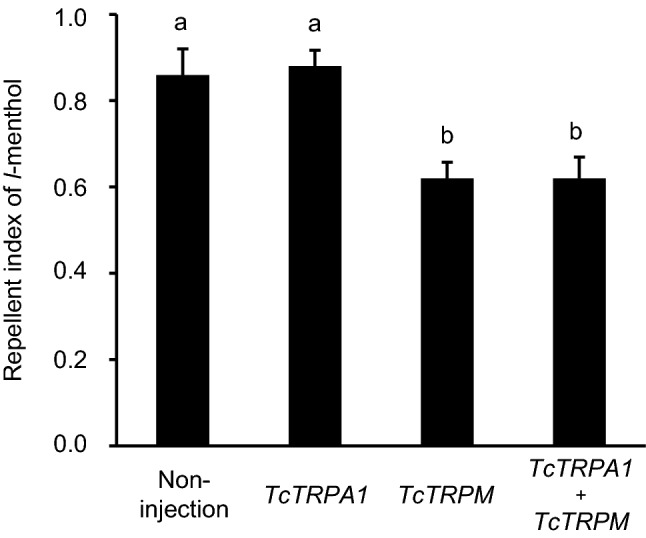
Comparison of TcTRPA1 mediation for *l*-menthol-based repellency behavior of *Tribolium castaneum* among dsRNA treatments of *TcTRPA1*, *TcTRPM*, and double *TcTRPA1* plus *TcTRPM*. Twenty beetles were released in the area-preference test, treated with 2.5 μmol /cm^2^ of *l*-menthol; five biological replications were performed. Data are expressed as mean ± standard error of the mean; the same letters above the bars indicate no significant difference at *P* > 0.05 (one-way ANOVA and Tukey–Kramer HSD tests). TRP: transient receptor potential.

TcTRPA1 intervention for *l*-menthol repellency was clarified using RNAi. The *TcTRPA1* dsRNA-treated beetles were not significantly different from the untreated beetles; however, the *TcTRPM* dsRNA-treated beetles and the double *TcTRPM* plus *TcTRPA1* dsRNA-treated beetles revealed a significantly reduced repellent index compared with the repellent index seen in the untreated beetles (*F*_3,16_ = 9.50, *P* < 0.001) (Fig. 5). There was no significant difference between *TcTRPM* and double *TcTRPM* plus *TcTRPA1* dsRNA treatments. The qRT-PCR analysis revealed that the *TcTRPA1* and the double *TcTRPA1* plus *TcTRPM* dsRNA treatments reduced the target *TcTRPA1* transcripts by approximately 20% (*F*_2,6_ = 216.67, *P* < 0.001), and the *TcTRPM* and the double *TcTRPM* plus *TcTRPA1* dsRNA treatments reduced the target *TcTRPM* transcripts by approximately 35% (*F*_2,6_ = 38.58, *P* < 0.001) (Supplementary Fig. S4).

## Discussion

The TRPA1 channel is widely expressed in insects, and TRPA1 is activated by noxious heat and irritant chemicals. To elucidate the molecular mechanism of citronellal aversion in *T. castaneum*, RNAi-based knockdown of *TcTRPA1* was employed and repellency movement was evaluated. The results clearly showed that the *TcTRPA1* dsRNA-treated beetles were not repelled by citronellal in the area-preference test, demonstrating the TcTRPA1-mediated repellency behavior for citronellal.

The synthetic repellent compound DEET, *N*,*N*-diethyl-3-methylbenzamide, remains the gold standard. DEET-based repellency involves multiple pathways and mechanisms; however, it remains an area of contention^30^. In our prior studies, *T. castaneum* showed repellency behavior for DEET^29,31^; however, to the best of our knowledge, there are no studies that report that DEET activates insect TRP channels. We analyzed the area-preference test with *TcTRPA1* dsRNA treatments for DEET repellency, and the repellent index of the *TcTRPA1* dsRNA-treated beetles did not show a significant difference from that of the untreated beetles, implying the specific interaction of citronellal against TcTRPA1.

The adult *TcTRPA1* transcript expression profile in the various body parts revealed that the expression in the antennae was dominant; a similar case was observed in the hemipteran order, in the brown planthopper *Nilaparvata lugens*, which showed high *NlTRPA1* expression in the antennae^32^. From these results, we proposed that *T. casteneum* repels citronellal with TcTRPA1-mediated antenna perception. We removed the antennae and compared the repellent index with that of the untreated beetles and showed a significant reduction in the repellent index by de-antennated treatment. Meanwhile, in our previous study, contact repellency activity of *l*-menthol did not show a significant difference between de-antennated and untreated beetles^29^, these results indicated antenna-expressed-TcTRPA1-mediated repellency behavior for citronellal.

In the case of dipteran species, two pathways: one is indirect contribution of TRPA1 in G protein/phospholicase C signaling cascade, and the other is direct Orco contribution, for citronellal avoidance was revealed in *D. melanogaster*^20,21^. In the oriental fruit fly *Bactrocera dorsalis*, it was demonstrated that *BdOrco* dsRNA treatment resulted in the reduction of oviposition repellents induced by citronellal^33^. In mosquitoes, citronellal binds directly to TRPA1 in *A. gambiae*^20^. In lepidopterans, the TRPA1 channel of *Helicoverpa armigera* revealed that in vitro heterologous expression of *Xenopus* oocyte functions not only as a thermal sensor but also as an irritant chemical sensor, including citronellal^34^.

In contrast, in the case of Hemiptera, the western tarnished plant bug *Lygus hesperus* showed that although *LhTRPA1* dsRNA injection reduced citronellal-induced avoidance movement, *LhOrco* dsRNA injection did not alter the response, suggesting an OR-independent pathway for citronellal repellency^35^. To clarify the OR-mediated citronellal repellency of coleopteran beetles, we next verified the TcOrco-mediated repellency for citronellal. The area-preference test revealed that *TcOrco* dsRNA-injected beetles did not show significant differences in repellency behavior from non-injected beetles. Since Orco is widely co-expressed with ORs and strongly conserved across insects^36^, the results suggested non-involvement of the OR perception system for citronellal perception in *T. casteneum*. In combination with these results, we propose that evolutionary diversity has arisen for citronellal perception in Insecta.

Multiple TRP channels have been suggested to mediate the perception of menthol^11,12^ . In *Drosophila* larvae, menthol evoked TRPM- and TRPA1-dependent nocifensive rolling behavior, which is required for multidendritic class IV nociceptor neurons^28^. In that analysis, homozygous mutants, each of TRPM and TRPA1, showed significantly fewer rolled larvae in response to menthol; furthermore, heterozygous mutants of both TRPM and TRPA1 showed significantly inhibited rolling behavior for menthol, indicating a genetic interaction in menthol sensing. In contrast, the *l*-menthol repellency behavior of adult *T. castaneum* was affected by TcTRPM. The relative expression levels of *TcTRPM* showed minimal differences among various adult tissues such as abdomen, thorax, head, leg, and antenna^29^. However, the *TcTRPA1* transcripts in the present study were predominantly expressed in the antennae, while the de-antennated adults still exhibited repellency behavior for *l*-menthol, suggesting that only TcTRPM was required for menthol-evoked repellency. In the case of mammals, except for the TRP channels, it has been revealed that menthol influences different kinds of voltage-gated ion channels and ligand-gated ion channels, such as GABA, glycine receptors, and nicotinic acetylcholine receptors^37^, suggesting the presence of different targets for the menthol-mediated repellency behavior in adult *T. castaneum*.

We describe the antennae-expressed TcTRPA1-mediated repellency behavior by which *T. castaneum* responds to citronellal, our results suggest that coleopteran TRPA1 represents a potential target for sustainable insect repellents. Intriguingly, Tian et al. recently reported that *D. melanogaster* TRPγ, contained in TRPC subfamily, was directly activated by citronellal to initiate repellent behavior^38^, for which further experiments and screening are desirable even in coleopteran beetles.

## Methods

### Insects

Laboratory colonies of *Tribolium castaneum* were used in the present study. They were fed on whole-grain wheat containing 5% dried yeast and reared at 28 °C under dark conditions in an incubator.

### Behavioral bioassay

Repellency behavior was evaluated using the area-preference method. Racemic citronellal was purchased from Kanto Chemical Co., Inc. (Tokyo, Japan) while *l*-menthol and *N*,*N*-diethyl-3-methylbenzamide (DEET) were purchased from Tokyo Chemical Industry Co., Ltd. (Tokyo, Japan). The test compounds were dissolved in acetone at concentrations of 10, 25, and 50 mg/mL for citronellal; 25 mg/mL for *l*-menthol; and 10 mg/mL for DEET. Filter papers 90 mm in diameter (ADVANTEC No.2, Toyo Roshi Kaisha Ltd., Tokyo, Japan) were cut in half, and 500 μL of the solution was uniformly applied to half of the filter paper using a micropipette. The other half was treated with 500 μL of acetone as a control. Both filter papers were air-dried to completely evaporate the solvent and carefully rebounded using tape. Each reassembled filter paper was placed in a 90 mm glass petri dish. Twenty adult beetles were released into the center of each filter paper disk, and the glass petri dish was then covered and maintained at 28 °C under dark conditions. Each test was replicated five times. After 3 h, the beetle positions in each area were counted. The repellency activity of the compound was expressed in terms of the repellent index (RI), calculated according to the following formula:

$${\text{Repellent}}\,{\text{ index}}\, \, \left( {{\text{RI}}} \right) \, = \, \left[ {{{\left( {N_{{\text{c}}} - N_{{\text{t}}} } \right)} \mathord{\left/ {\vphantom {{\left( {N_{{\text{c}}} N_{{\text{t}}} } \right)} {\left( {N_{{\text{c}}} N_{{\text{t}}} } \right)}}} \right. \kern-\nulldelimiterspace} {\left( {N_{{\text{c}}} + N_{{\text{t}}} } \right)}}} \right]$$

where *N*_c_ is the number of beetles present in the control (solvent alone) area and *N*_t_ is the number of beetles present in the treated area.

### cDNA cloning

The three adult *T. castaneum* were crushed together using a Biomasher II (Nippi Inc., Tokyo, Japan). Total RNA was extracted using the ReliaPrep RNA Cell Miniprep System (Promega Corporation, Madison, WI, USA), and first-strand complementary DNA (cDNA) was generated using ReverTra Ace α (TOYOBO Co., Ltd., Osaka, Japan). Full-length *TcTRPA1* and *TcOrco* coding sequences were amplified by PCR using PrimeSTAR GXL DNA Polymerase (Takara Bio Inc., Shiga, Japan) for *TcTRPA1* and KOD plus Neo (TOYOBO) for *TcOrco* with the specific primers designed from the sequence data obtained from iBeetle-Base^39^ (Supplementary Table S1). PCR was performed on a T100 thermal cycler (Bio-Rad, Hercules, CA, USA) under the following conditions: 35 cycles at 98 °C for 10 s, 58 °C for 15 s, and 68 °C for 4 min for *TcTRPA1*; 94 °C for 2 min, followed by 40 cycles at 98 °C for 10 s, 58 °C for 30 s, and 68 °C for 2 min for *TcOrco*. The PCR products were electrophoresed at 0.8% for *TcTRPA1* and 1% for *TcOrco* agarose-gel, then purified using a MiniElute Gel Extraction Kit (QIAGEN, Hilden, Germany). The PCR product was assembled with the pUC19 plasmid vector using the In-Fusion HD cloning kit (Takara Bio) and transformed into competent DH5α *Escherichia coli* cells (ECOS Competent *E. coli* DH5α, NIPPON GENE, Tokyo, Japan). Plasmid DNA was purified using a FastGene Plasmid Mini kit (NIPPON Genetics, Tokyo, Japan) and sequenced. BlastP search was performed using TcTRPA1 amino acids sequence, and maximum-likelihood (ML) phylogenetic analysis for the aligned sequence and the ML phylogenetic tree was constructed using MEGA X^40^. Bootstrap support was obtained using 1,000 bootstrap replications.

### RNAi-mediated gene knockdown

The *TcTRPA1* and *TcOrco* gene templates containing the T7 promoter sequence at the 5′ end were amplified with primers (Supplementary Table S1) using KOD Plus Neo (TOYOBO) under the following conditions: 94 °C for 2 min, 40 cycles at 98 °C for 10 s, 58 °C for 30 s, and 68 °C for 1 min. The amplified templates were used for the synthesis of dsRNA using the RiboMAX Large-Scale RNA Production System T7 (Promega). The dsRNA products were quantified using a NanoDrop spectrophotometer (Thermo Fisher Scientific, Inc., Wilmington, DE, USA). *EGFP* dsRNA was synthesized from the pEGFP-C1 plasmid (Takara Bio USA, Inc., Mountain View, CA, USA) for the control, and *TcTRPM* dsRNA was prepared in the same manner as previously reported^29^. All dsRNA concentrations were adjusted to 2000 ng/μL using RNase-free water and injected into the pupal stage using a glass needle mounted with a micromanipulator (Narishige, Tokyo, Japan). After injection, the pupae were incubated in a 24-well plate set on a filter paper disk (ADVANTEC No.2) with a piece of whole-grain wheat with 5% of dried yeast at the bottom and incubated at 28 °C. After emergence, approximately 7-day-old adults were used for the area-preference test and subsequent qRT-PCR.

### Quantitative analysis of transcription

To clarify RNAi-knockdown efficiency, total RNA was extracted from three adults after the area-preference test, and to compare relative expression level, total RNAs of each tissue (300 antennae, 30 heads, 20 thoraxes, 40 legs and four abdomens) from approximately 7-day-old adults were extracted in a similar manner, and cDNA was generated using ReverTra Ace qPCR RT kit (TOYOBO). Gene-specific primers for qRT-PCR were designed using Primer3 (Supplementary Table S1). qRT-PCR was performed on a StepOnePlus Real-Time PCR System (Applied Biosystems, Foster City, CA, USA) and THUNDERBIRD Next SYBR qPCR Mix (TOYOBO). The PCR amplification program consisted of 95 °C for 30 s, followed by 45 cycles at 95 °C for 5 s and 60 °C for 30 s, and a dissociation step (95 °C for 15 s, 60 °C for 30 s, and 95 °C for 15 s) for melting curve analysis. The expression results were analyzed using the ∆∆Ct method. *TcRPS6* gene (encoding ribosomal protein S6) was used to normalize gene expression, and mean Ct values for each gene were obtained from three replicates.

## Statistical analysis

In the area-preference tests, the mean number of beetles in the sample area was compared with the number of beetles in the control area using a two-tailed paired *t*-test. The relative expression levels of the transcripts for dsRNA-treated beetles and each tissue, and the RI values were compared by one-way ANOVA, followed by the Tukey–Kramer HSD test. The RI values of antennae-dissected beetles, *TcOrco* dsRNA-treated beetles against citronellal, and *TcTRPA1* dsRNA-treated beetles against DEET were compared using a two-tailed student’s *t*-test. All statistical analyses were performed using R software.

## Supplementary Information

Supplementary Information.

## Data Availability

All data generated or analyzed during this study are included in this article and its supplementary information files.
