# Supplementary material for: TRPA1-mediated repellency behavior in the red flour beetle Tribolium castaneum
Source: Sci Rep. 2022 Sep 10;12:15270. doi: 10.1038/s41598-022-19580-z (PMC9464225; doi:10.1038/s41598-022-19580-z)
Supplement: Supplementary file 1 — Supplementary Information. [file 41598_2022_19580_MOESM1_ESM.pdf]

Supporting information

Title:

TRPA1-mediated repellency behavior in the red flour beetle *Tribolium castaneum*

Author names and affiliations:

Kenji Shimomura[1,*], Soshi Ino[1], Kazuya Tamura[1], Takehito Terajima[1], Motohiro Tomizawa[1]

[1]Department of Chemistry for Life Sciences and Agriculture, Tokyo University of Agriculture, Sakuragaoka 1-1-1, Setagaya-ku, Tokyo 156–8502, Japan

*corresponding. k3shimom@nodai.ac.jp

Supplementary table S1. Primer sequences used in the present study

| Gene | Accession No. | | Primer (5' -> 3' ) | Size (bp) |
|---|---|---|---|---|
| PCR | | | | |
| *TcTRPA1* | TC032665 | Forward | ATGCCAAATCTAATGCATCTCC | 3729 |
| | | Reverse | CTAAGTGCTGCTGCCTTTATTG | |
| *TcOrco* | TC015127 | Forward | ATGATGAAATTCAAGGTCACGG | 1428 |
| | | Reverse | TCATTTGAGTTGCACCAACAC | |
| dsRNA | | | | |
| *TcTRPA1* | | Forward | TAATACGACTCACTATAGGGTGCTCACAGTTTTACGCCGA | 523 |
| | | Reverse | TAATACGACTCACTATAGGGTGGTGCCCCAAAAAATCGGAA | |
| *TcOrco* | | Forward | TAATACGACTCACTATAGGGTCTGTTCGTGGCTGATTTTCGC | 314 |
| | | Reverse | TAATACGACTCACTATAGGGCGACCTGACCATCAACTCCTGT | |
| qRT-PCR | | | | |
| *TcTRPA1* | | Forward | TGGATGCGGTGGATTTTCTCCT | 108 |
| | | Reverse | GCACGGACACCTTGTTAAGCTC | |
| *TcOrco* | | Forward | TTGGTCAAGGCATGGTACCCAT | 143 |
| | | Reverse | GCGAAAATCAGCCACGAACAGA | |

Underline represents the T7 promoter sequence.

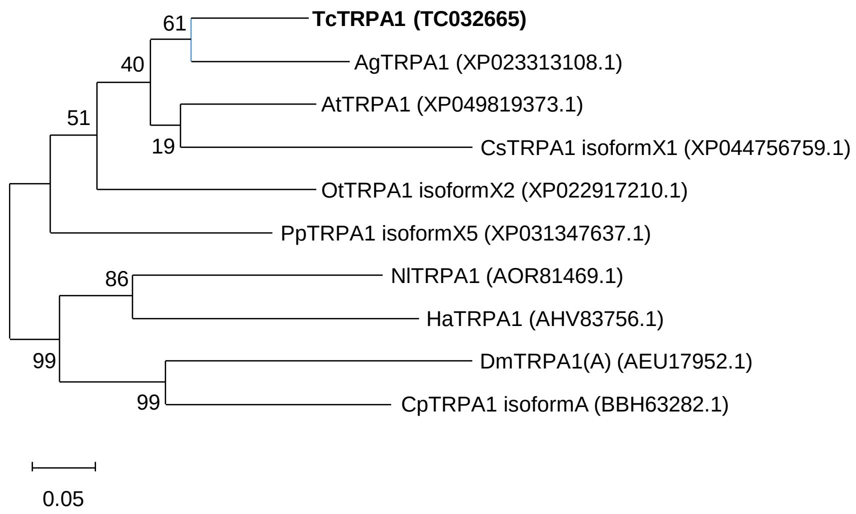


Supplementary figure S1. Maximum-likelihood phylogenetic tree of *Tribolium castaneum* TRPA1 with insect TRPA1 sequences, *Anoplophora glabripennis* (Ag), *Aethina tumida* (At), *Coccinella septempunctata* (Cs), *Onthophagus taurus* (Ot), *Photinus pyralis* (Pp), *Nilaparvata lugens* (Nl), *Helicoverpa armigera* (Ha), *Drosophila melanogaster* (Dm), and *Culex pipiens* (Cp). Bootstrap values are indicated on branches, and rate of amino- acid substitutions per site is shown in the scale bar.

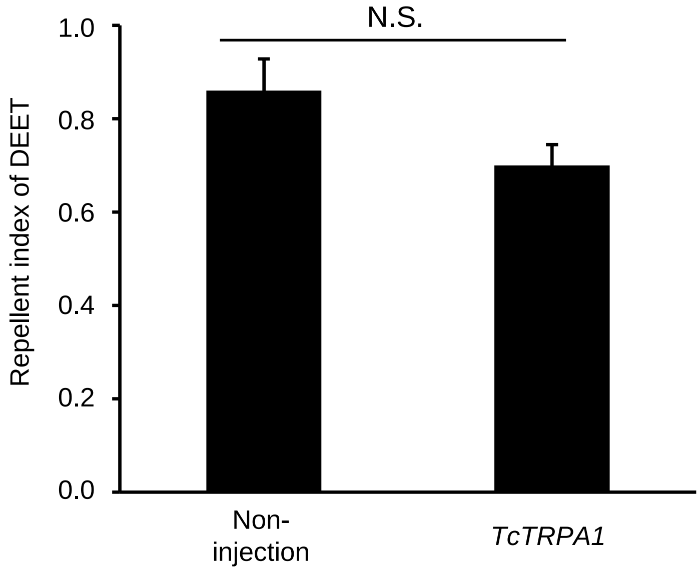


Supplementary figure S2. TcTRPA1 did not mediate the DEET-based repellency behavior of *Tribolium castaneum*. Twenty beetles were released in the area-preference test, treated with 0.8 μmol / $cm^2$ of DEET; five biological replications were performed. Data are expressed as mean ± standard error of the mean, N.S. indicates that there was no significant difference between non-injected and *TcTRPA1* dsRNA-treated beetles ($P$ = 0.084, two-tailed student's t-test).

TRP: transient receptor potential; DEET: *N*,*N*-diethyl-3-methylbenzamide

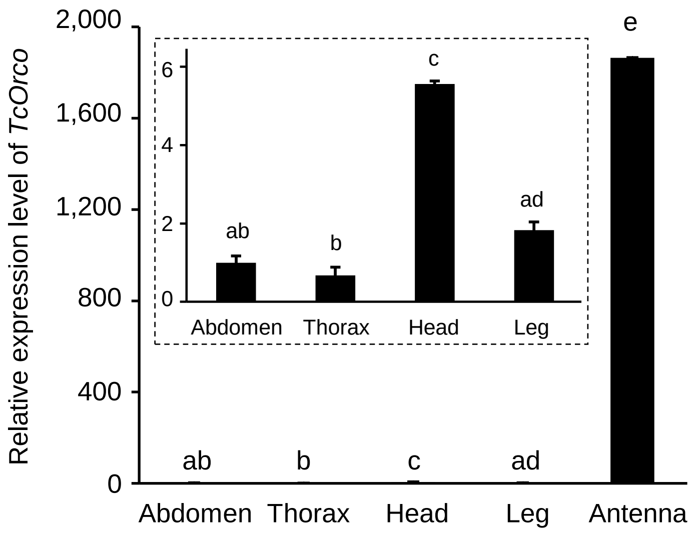


Supplementary figure S3. Tissue expression profile of *TcOrco*. Relative expression levels of *TcOrco* transcripts in the abdomen, thorax, head, leg, and antenna of adult *Tribolium castaneum* were revealed as fold changes relative to the corresponding expression levels seen in the abdomen (n = 3) analyzed by qRT-PCR. Data are expressed as mean ± standard error of the mean. The same letters above the bars indicate no significant difference at $P > 0.05$ (one-way ANOVA and Tukey–Kramer HSD tests). Inlet: Focusing on a low level of relative expression.

Orco: odorant receptor co-receptor

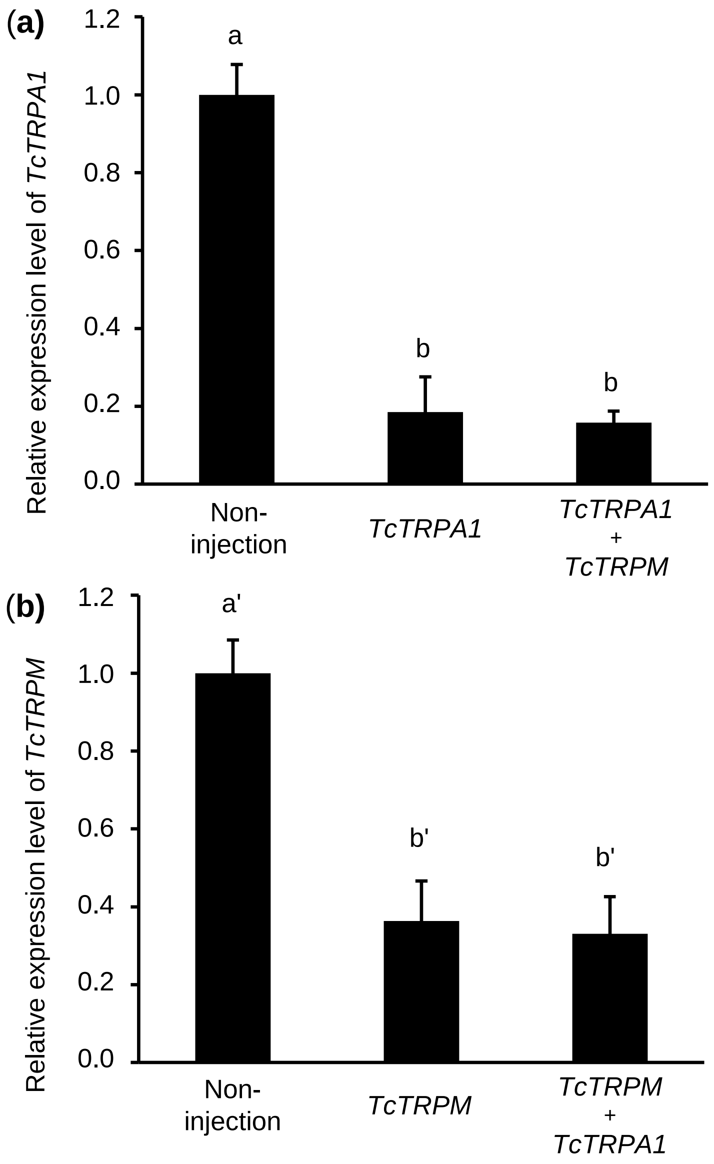


Supplementary figure S4. RNAi-mediated gene knockdown. (**a**) Relative expression levels of *TcTRPA1* transcripts when *TcTRPA1* and double *TcTRPA1* plus *TcTRPM* dsRNA were micro-injected. (**b**) Relative expression levels of *TcTRPM* transcripts when *TcTRPM* and *TcTRPM* plus *TcTRPA1* dsRNA were micro-injected. After adult emergence, the targeted transcripts were measured using qRT-PCR. The expression level was revealed as fold change relative to the expression level seen in the non-injected beetles (n = 3). Data are expressed as mean ± standard error of the mean. The same letters above the bars indicate no significant difference at $P > 0.05$ (one-way ANOVA and Tukey–Kramer HSD tests).

TRP: transient receptor potential
